# Letter from the Editor in Chief

**DOI:** 10.19102/icrm.2020.110702

**Published:** 2020-07-15

**Authors:** Moussa Mansour


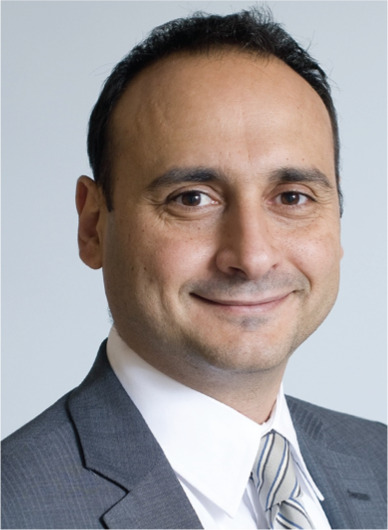


Dear Reader,

Circumferential pulmonary vein (PV) isolation has become the main treatment for paroxysmal atrial fibrillation (AF). However, the clinical success rate observed with this approach alone has not been matched to date in patients with persistent AF. It has been hypothesized that triggers/drivers for AF outside of the PVs are responsible for the lower success rate observed among patients with persistent AF after PV isolation. Over the past 10 years, multiple studies have sought to identify and ablate non-PV targets including complex fractionated atrial electrograms, rotors, ganglionated plexi, areas of fibrosis, and isoproterenol-induced focal activity. While most of these studies failed to demonstrate a benefit with the ablation of the first three mentioned targets, the ablation of areas of fibrosis and isoproterenol-induced focal activity, respectively, seems to show a significant level of benefit when performed in conjunction with PV isolation.

In particular, the ablation of areas of fibrosis has been gaining momentum and is currently been tested in the Efficacy of Delayed-enhancement Magnetic Resonance Imaging–guided Ablation Versus Conventional Catheter Ablation of AF (DECAAF II) randomized clinical trial. This issue of *The Journal of Innovations in Cardiac Rhythm Management* contains an important manuscript titled “Advancements in Imaging for Atrial Fibrillation Ablation: Is There a Potential to Improve Procedural Outcomes?”^1^ by Drs. Obeng-Gyimah and Nazarian. In this paper, the authors describe different applications for left atrial imaging performed before, during, and after ablation. Among the techniques mentioned, I believe that preprocedural left atrial imaging for the identification of fibrotic areas is the most important.

The accurate identification of areas of scar using currently available imaging technologies is difficult: it relies on the use of gadolinium enhancement to delineate scar, with universal criteria to clearly separate healthy from sick atrial tissue inadequately established. As a result, the outcomes of imaging studies have been variable and heavily dependent on both the operator and center involved. The development of advanced imaging techniques using contrast agents that delineate scar area in a more reliable way is of the utmost importance to enable scar imaging to be performed at a larger scale.

I hope that you enjoy reading this issue of the journal.

Sincerely,


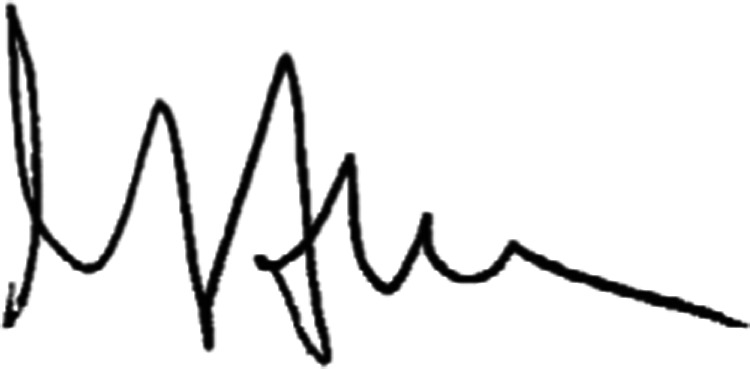


Moussa Mansour, md, fhrs, facc

Editor in Chief

The Journal of Innovations in Cardiac Rhythm Management

MMansour@InnovationsInCRM.com

Director, Atrial Fibrillation Program

Jeremy Ruskin and Dan Starks Endowed Chair in Cardiology

Massachusetts General Hospital

Boston, MA 02114
